#  Cytotoxicity of ICD-85 NPs on Human Cervical Carcinoma HeLa Cells through Caspase-8 Mediated Pathway 

**Published:** 2013

**Authors:** Saeed Moradhaseli, Abbas Zare Mirakabadi, Ali Sarzaeem, Morteza Kamalzadeh, Reza Haji Hosseini

**Affiliations:** a *Department of Biochemistry, Faculty of Basic Sciences, Payame Noor University, Tehran, Iran. *; b *Department of Venomous Animals and Antivenom Production, Razi Vaccine and Serum Research Institute, Karaj, Iran.*; c *Department of Quality control, Razi Vaccine and Serum Research Institute, Karaj, Iran. *

**Keywords:** Cancer, Nanoparticles, ICD-85, HeLa cell line, MTT assay, Caspase-8

## Abstract

The biological application of nanoparticles (NPs) is a rapidly developing area of nanotechnology that raises new possibilities in the treatment of human cancers. The cytotoxicity was evaluated by MTT and LDH assays. The apoptotic effect of free ICD-85 and ICD-85 NPs on HeLa cells was assessed using caspase-8 colorimetric assay. The MTT assay showed that ICD-85 NPs could enhance the *in-vitro *cytotoxicity against HeLa cells compared to the free ICD-85. The IC_50 _value at 72 h was reduced from 25 ± 2.9 μg/mL for free ICD-85 to 15.5 ± 2.4 μg/mL for ICD-85 NPs. However, LDH assay demonstrated that ICD-85 has dose-dependent cytotoxicity on HeLa cells while ICD-85 NPs exhibited weaker cytotoxicity on same cells. The results also indicate that ICD-85-induced apoptosis on HeLa cells is associated with the activation of caspase-8. Moreover, caspase-8 assay analysis demonstrated that the ICD- 85 NPs induced a higher apoptotic rate in HeLa cells compared to free ICD-85. Our results demonstrated that the encapsulation of ICD-85 enhances its anti-proliferative effects. Taken together, these results suggest that the delivery of ICD-85 in nanoparticles may be a promising approach for the treatment of the cancer.

## Introduction

Cancer is the most distressing and life-threatening disease that enforces severe death worldwide. Mortality is still unacceptably high despite many therapeutic advances (1, 2). Nowadays, there are four standard methods for the treatment of cancer: surgery, chemotherapy, radiation therapy, and immunotherapy (3). The most common option used for treatment of cancer is chemotherapy but it is often associated with the number of drawbacks, *i.e*. nonselective distribution of drugs, multidrug resistance, enhanced drug toxicity, undesirable side effect to normal tissue and inherent lacking of beneficial response of cytotoxic anti-cancer drug (4-6). Thus, we need to focus on the development of new drugs having potent anti-cancer effect and lower side effect. Great interest is currently being paid to natural products for their interesting anti-cancer activities (7, 8). Venom of some animals such as snake and scorpion had been reported to be cytotoxic on tumor cells which were mediated through inducing apoptosis in the target cells (9-11). The application of nanotechnology to drug delivery has already had a significant impact on many areas of medicine and change the scale and methods of drug delivery (12). Nanoparticles have been investigated for the delivery of different types of therapeutic agents including proteins, peptides and DNA (13, 14). Nanoparticles can protect the encapsulated agent from enzymatic degradation (15). Among the different carriers for controlled drug delivery, there has been rising interest in nano-sized self-aggregates composed of natural polysaccharides such as curdlan (16), dextran (17), alginate (18) and chitosan (19).

Alginate is a naturally occurring, water-soluble, linear unbranched polysaccharide extracted from brown seaweed. It consists of D-mannuronate and L-guluronate residues, which are arranged in both homopolymeric and heteropolymeric blocks. Alginate has been reported as mucoadhesive, biocompatible, non-immunogenic substance which undergoes dissolution and biodegradation under normal physiological conditions (20, 21).

Our previous studies revealed an inhibitory effect of ICD-85 (venom-derived peptides) on breast cancer cell line MDA-MB231 (22). ICD-85 was also confirmed by *in-vivo *studies to suppress the breast tumor in mice (23). In this report we employed polymer-based nanoparticle approach to improve upon its effectiveness. The aim of the present study was to evaluate the anti-proliferative activity of ICD-85 NPs relative to free ICD-85 *in-vitro*.

## Experimental

*Materials*

The cell culture medium (DMEM), fetal bovine serum (FBS), Trypsin-EDTA, penicillin and streptomycin were provided by Gibco (USA). Human cervical carcinoma HeLa cells were obtained from Razi Vaccine and Serum Research Institute cell bank *(*Karaj, Iran*). *Sodium alginate and poly-L-lysin were purchased from Sigma-Aldrich Chemical (Germany). 3-(4,5-dimethyl-thiazol-2-yl)-2,5-diphenyltetrazolium bromide (MTT), calcium chloride and dimethyl sulfoxide (DMSO) were purchased from Merck (Darmstadt, Germany).

*ICD-85 (venom derived peptides)*

The active fraction of ICD-85 is a combination of three peptides, ranging from 10,000 to 30,000 Da, derived from the venoms of an Iranian brown snake (*Agkistrodon halys*) and a yellow scorpion (*Hemiscorpius lepturus*). This fraction was formulated and provided by the corresponding author. The ICD-85 peptides were selected based on a study of crude venom cytotoxicity. The crude venom showed antigrowth activity on the MDA-MB231 and HL-60 cell lines. Then, the venoms were fractionated; the active peptides were isolated and subsequentially tested on the same cell line (22, 23).

*Preparation of ICD-85 NPs and particle size*

ICD-85 NPs was prepared by the ionic-gelation method (24). Initially, sodium alginate was dissolved in distilled water at 3 mg/mL. Then, a solution of calcium chloride at 1 mg/mL was prepared. Finally, 5 mL of the sodium alginate solution was added dropwise under constant stirring to 2 mL calcium chloride solution. Nanoparticles were separated by centrifuging (Ependorf, Germany) at 13,000 rpm at 14°C for 30 min, freeze-dried, and stored at 4-8°C. The ICD-85 loading nanoparticles were prepared with incorporation of sodium alginate solution, into calcium chloride solution containing 500 μg/mL of ICD-85. The mean particle size of the obtained ICD-85 NPs was 200 ± 11.5 nm, as measured by Zetasizer (SEM-Tech, USA).

*Cell culture*

The HeLa cell line was cultured in the DMEM medium, supplemented with FBS (10%), penicillin (100 Units/mL) and streptomycin (100 μg/mL). The cells were grown in CO_2_ incubator (Memmert, Germany) at 37°C with 90% humidity and 5% CO_2_. Cells were subcultured regularly using trypsin/EDTA (25).

*Determination of antiproliferative activity*

The antiproliferative effects of ICD-85 and ICD-85 NPs were measured using the MTT colorimetric assay (26). The cells were plated at a density of 5000 cells/well and seeded in 96-well plates (Nunc, Denmark). After 24 h, the cells were treated with different concentrations of ICD-85 and ICD-85 NPs for 72 h. After the treatment, media were carefully removed. Cells were washed twice with PBS before that the 100 μL medium with 20 μL of MTT solution (5 mg/mL in PBS) was added to each well. The plate was incubated at 37°C for 4 h. Then, the medium was totally removed and 200 μL DMSO was added to each well. DMSO was also added to the wells designated as reference blanks. The plate was vibrated for 15 min. The absorbance, which was proportional to cell viability, was subsequently measured at 570 nm in each well using an ELISA plate reader (Dynex MRX II, USA). Cytotoxicity was expressed as a percentage of growth inhibition, relative to untreated cultures, and the concentration required to inhibit the cell growth by 50% (IC_50_) was calculated. Percentage growth inhibition was equal to [1-(OD of treated/OD of control)] ×100. Each experiment was performed using six replicates for each drug concentration and repeated in triplicate.

*LDH assay*

Toxicity was assayed by measuring the activity of the cytosolic enzyme, lactate dehydrogenase (LDH), released into the culture medium after the membrane damage (27). Samples from clarified medium of treated and untreated control wells were taken after 24 h of incubation and the LDH activity was measured using the cytotoxicity assay, CytoTox 96® (Promega, USA) associated with a fully automated microplate reader photometer (BioTek, USA).

*Colorimetric estimation of caspase activation*

The extent of caspase activation in HeLa cells treated with ICD-85 and ICD-85 NPs was assessed using commercially available colorimetric assay kit in accordance with the protocol supplied by the manufacturer (BioVision, USA). Briefly, cells were cultured (106 Cells/mL) in 25 cm^2^ flasks (Nunc, Denmark), with total volume of 5 mL medium per flask and incubated with ICD-85 and ICD-85 NPs for 24 h. At the end of the treatment, cell pellet was lysed by the addition of lysis buffer supplied with the kit. The cell lysates were added to the 96-well plates (Nunc, Denmark) and incubated with caspase-8 substrate at 37°C for 2 h. Absorbance in wells was measured at 405 nm. The increase in the activity of caspase-8 was determined by comparing these results with the levels in untreated controls.

*Light microscopy*

For these experiments, the HeLa cells were treated with 28 μg/mL ICD-85 and ICD-85 NPs in six-well transparent plates (Nunc, Denmark). After 24 h of exposure, the cell morphology was examined under an inverted light microscope (Olympus CK2, Japan).

*Statistical analysis*

Experiments were carried out at least in triplicate and results were expressed as mean ± SD. Statistical analysis of the differences in the measured properties of the groups were performed with one-way analysis of variance and the determination of confidence intervals, with the statistical package (SPSS version 18). In all cases, p < 0.05 was considered statistically significant.

## Results

*In-vitro cytotoxicity studies*

**Figure 1:**
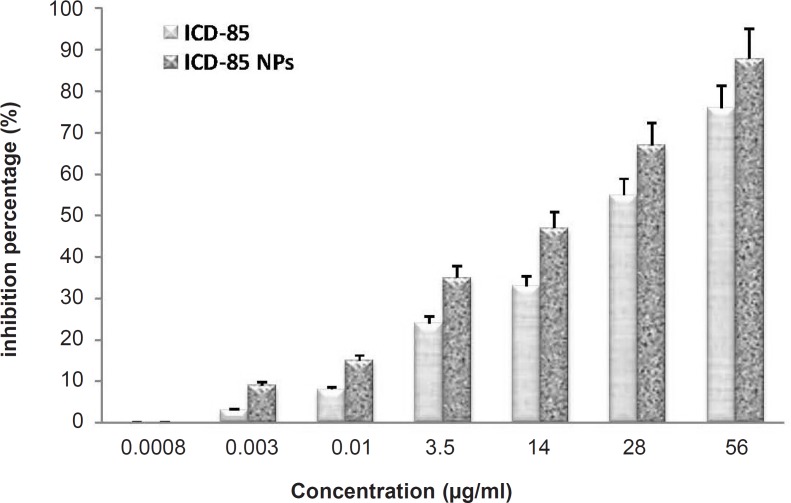
Comparison of Inhibitory effect of ICD-85 and ICD-85 NPs on HeLa cells: Cell proliferation was determined by the MTT method. The concentrations of the free ICD-85 and ICD-85 NPs were calculated to be equal. Proliferation of untreated cells (0 μg) was taken as 100%. The measurements of the treated cells were normalized to the control measurement (100%). All measurements are reported as mean ± SD (n = 3, p < 0.05).

The cytotoxicity of free ICD-85 and ICD-85 NPs on the human cervical carcinoma HeLa cells was determined by the MTT method for cell proliferation. The concentrations of ICD-85 NPs treatments were calculated in such a way to include equivalent amounts as the ICD-85 treatments. The cells were treated with various concentrations of free ICD-85 and ICD-85 NPs at 37°C for 72 h. All measurements were normalized to the measurement of the control untreated cells which was considered to be 100%. Our initial observation was that the treatment of cells with ICD-85 concentrations less than 0.003 μg/mL did not affect the cell proliferation activity. ICD-85 NPs were found to be more toxic to human cervical carcinoma HeLa cells than the free drug: extrapolation from the dose-response curve demonstrates that a dose of 28 μg/mL of free ICD-85 inhibited 55% of the cell population after 72 h, whereas a dose of 28 μg/mL ICD-85 NPs inhibited nearly 70% of the population. These concentrations indicate that the ICD-85 NPs were more potent than the free ICD-85 for the same concentration (Figure 1). The IC_50 _was determined to be about 25 ± 2.9 μg/mL for ICD-85 and 15.5 ± 2.4 μg/mL for ICD-85 NPs.

*LDH release*

**Figure 2:**
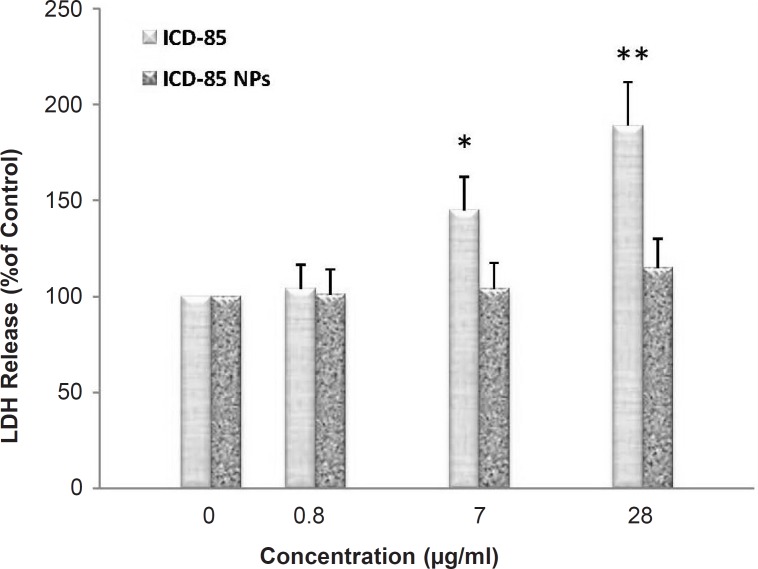
Percentage of LDH released from HeLa cells following 24 h exposure to increasing concentrations of ICD-85 and ICD-85 NPs. LDH released from untreated cells (0 μg) was taken as 100%. Results are expressed as percentage control values ± SD of three or more independent experiments (*p < 0.05, **p < 0.01 relative to control).

ICD-85-induced cell membrane damage was assessed by the LDH release assay. After 24 h, treatment with ICD-85 resulted in a significant increase in LDH release relative to the untreated cells (Figure 2). After 24 h of treatment with 28 μg/mL ICD-85, LDH release was increased to 189 ± 29.4% of control values (p < 0.01). Treatment with 7 μg/mL ICD-85 the increased LDH release to 145 ± 23% of control values (p < 0.05), whereas treatment with 0.8 μg/mL ICD-85 reduced this amount to 104 ± 17% of control values. In contrast, LDH determination of cultured media of human cervical carcinoma HeLa cells exposed to various concentrations of ICD-85 NPs revealed that no significant change occurred as compared with untreated cells. 

*ICD-85 and ICD-85 NPs-induced apoptosis in HeLa cells*

**Figure 3:**
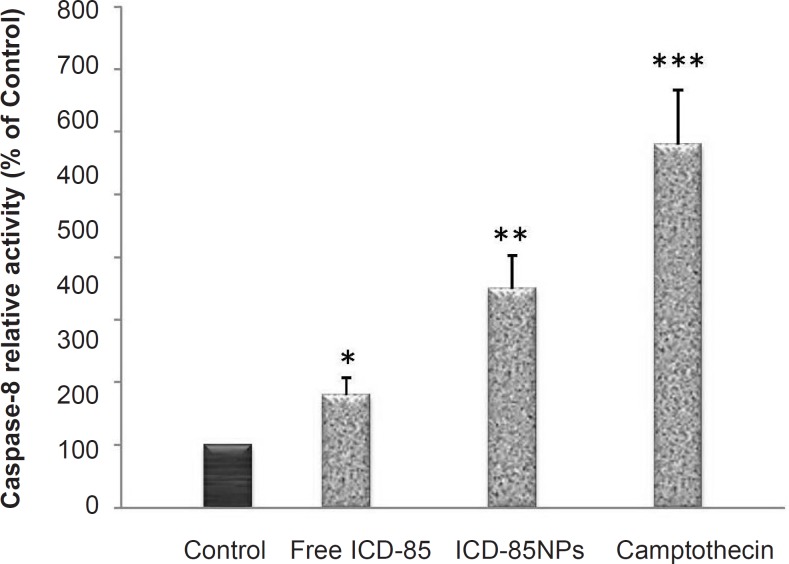
Effects of ICD-85 and ICD-85 NPs on activity of caspase-8 in HeLa cells: Caspase-8 activity in HeLa cells following 24 h of incubation with 28 μg/mL ICD-85 and ICD-85 NPs. Results are expressed as percentage control values ± SD of three or more independent experiments. Camptothecin was used as a positive control of caspase-8 activity (*p < 0.05, **p < 0.01, ***p < 0.001 relative to control).

To evaluate the molecular effectors pathway of apoptosis induced by ICD-85 and ICD-85 NPs, the activity of caspase-8 was measured using caspase-8 assay kit (BioVision, USA) after 28 μg/mL ICD-85 and ICD-85 NPs were added to HeLa cells for 24 h. As shown in Figure 3, the activation of caspase-8 was significant in ICD-85 (p < 0.05) and ICD- 85 NPs (p < 0.01) treated cells. As expected, treatment with 4 mM camptothecin, which was included as a positive control, produced a significant (p < 0.001) rise in caspase-8 activity. These results suggest that ICD-85 and ICD-85 NPs-induced apoptosis of human cervical carcinoma HeLa cells are associated with the activation of caspase-8.

*ICD-85 and ICD-85 NPs alters cell morphology*

**Figure 4:**
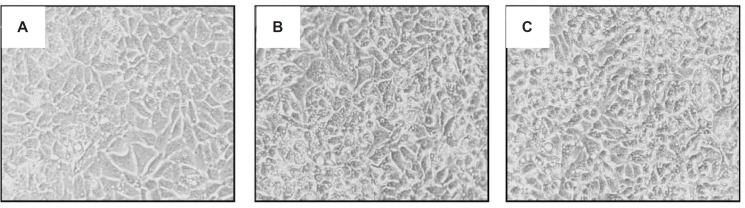
Morphological alterations of HeLa cells exposed to ICD-85 and ICD-85 NPs in cell culture medium for 24 h. (A) Micrograph of control cells (B) HeLa cells treated with 28 μg/mL of ICD-85 (C) HeLa cells treated with 28 μg/mL of ICD-85 NPs

The analysis of human cervix carcinoma cells (HeLa) treated and untreated cells revealed several morphological changes. Control cells exhibited a typical morphology after 24 h in culture (Figure 4A). In contrast, treated cells with 28 μg/mL ICD-85 and ICD-85 NPs showed destabilization of the plasma membrane, indicating an increasing progression toward cell death (Figures 4B, C).

## Discussion

Much of the contemporary research in the development of anti-cancer therapeutics from animal venoms has been focused on investigating the molecular mechanism by which an agent induces cytotoxicity and apoptosis in cancer cells (9-11). Some of proteins and peptides originated from animal venoms, when isolated, may bind specifically to the cancer cell membranes, affecting the migration and proliferation of these cells (28). Chlorotoxin is derived from scorpion venom, targeting metalloproteinases of glioma cells, leading to cell death (29).

ICD-85 used in this study which is the combination of three peptides, is derived from snake and scorpion venoms and seems to work synergistically to suppress the growth of cancer cells (22, 23). In the present work, we examined the cytotoxic effect of ICD-85 on the human cervical carcinoma HeLa cells. HeLa cell line was selected as the candidate in the present study, as this cell line is robust and show good growth characteristics, which include a short doubling time and a low requirement for growth factors (30). The human cervical carcinoma HeLa cell is one of the most often used models for cytotoxic studies (31, 32). Moreover, several studies have shown that this cell line is particularly resistant to cell death via apoptosis by a variety of different cytotoxic agents (33-35).

Our study clearly demonstrated that ICD-85 is cytotoxic to HeLa cells, showing a 72 h-IC_50 _of 25 ± 2.9 μg/mL. The MTT assay has been used in many experiments for the assessment of cytotoxic effects of tested agents. In this method, the MTT dye is reduced by living cells and this reaction is used as the end-point in a rapid drug-screening assay (26). Recently, we also reported that ICD-85 is cytotoxic to human leukemia (HL-60) cells, showing a 24 h-IC_50_ of 0.04 ± 0.015 μg/mL (36). These results confirm the cytotoxic effect of ICD-85 on cancer cells and indicate that the HeLa cells are less sensitive to ICD-85 toxicity than human leukemia (HL-60) cells. Treatment of HeLa cells with ICD-85 induced the morphological changes that confirm the MTT assay by changing in morphological appearance including rounding of cells which indicates the increase in apoptotic cell population (37). On the other hand, the elevation in the activity of caspase-8 observed in the present study suggests the apoptosis of cancer cells induced by ICD-85. Apoptosis is modulated by anti-apoptotic and pro-apoptotic effectors that involve a large number of proteins. The two main pathways for activating caspases are the death receptor and mitochondria-mediated mechanisms (38). Caspase-3 is a key executioner caspase involved in apoptosis, and its activity is controlled by upstream regulators, such as caspase-8 and caspase-9, which modulate the mitochondria- and death-receptor-dependent pathway, respectively (39).

Since the molecular weight of three peptides of ICD-85 is higher than the time they would penetrate the cell membrane of HeLa cells, the apoptosis might be exerted via the membrane receptors on cell surface. Therefore, to gain insight into how ICD-85 may affect the mechanisms of controlling apoptosis, we investigated the effect of ICD-85 on activating the extrinsic apoptotic pathway increase in caspase-8 activity.

In order to evaluate and compare the anti-proliferative activity of ICD-85 NPs on HeLa cells, the inhibitory effects of ICD-85 NPs versus free form of ICD-85 were studied. To prepare ICD-85 NPs, we used alginate. Alginate has been reported as a mucoadhesive, biocompatible, non-immunogenic substance that undergoes dissolution and biodegradation under normal physiological conditions (20, 21). Results revealed that ICD-85 NPs exhibited stronger growth inhibitory effect on HeLa cells compared with free ICD-85 (Figure 1). The amount of ICD-85 required to achieve IC_50_ of HeLa cell at 72 h exposure was lower than free form of ICD-85. The IC_50 _value for free ICD-85 was approximated to be 25 ± 2.9 μg/mL, whereas the IC_50_ for ICD-85 NPs was 15.5 ± 2.4 μg/mL. Almost all types of nanoparticles including polymeric nanoparticles (40), nanocrystals (41), dendrimers (42) and carbon nanotubes (43) have been evaluated for their suitability as multifunctional nanoparticles that can be applied for treatment of cancers. However, the exact mechanisms by which ICD-85 NPs exert their cytotoxic effects in cancer cells remain unclear. It is reported that the drug-encapsulated which controls releasing nanoparticles have the potential to improve current cancer chemotherapies by increasing drug efficacy, lowering drug toxicity, and maintaining a relatively high concentration of drug at the site of interest (14, 44). Moreover, *in-vivo *studies revealed that polymeric nanoparticle-bound anti-tumor agents prolong drug retention in tumors, reduce the tumor growth and increase the survival of tumor-bearing animals (45, 46).

The LDH activities of free ICD-85 and ICD-85 NPs were compared for HeLa cells. Although results of MTT assay clearly showed that ICD-85 NPs is comparatively more cytotoxic than free ICD-85 to HeLa cells, LDH activity determination showed that ICD-85 NPs exhibited weaker cytotoxicity (Figure 3). These results might be due to the sustained release properties of ICD-85 NPs which reduces the necrotic effect of ICD-85 while increasing apoptotic induction of it. This is in accordance with the caspase-8 assay analysis that demonstrated the ICD-85 NPs induced a higher apoptotic rate in HeLa cells compared to free ICD-85.

## Conclusion

Based on the findings of this work, it can be concluded that ICD-85 is cytotoxic to human cervical carcinoma HeLa cells through extracellular induction of apoptosis and ICD-85 NPs versus free form of ICD-85 can be more effective in growth inhibition of cells. However, the cytotoxicity of both forms (free and NPs) of ICD-85 on normal cells is under investigation in our laboratory and we hope that the toxicity of ICD-85 NPs on normal cells get reduced significantly in comparison with free ICD-85.
